# Comparison of antibacterial effects of a carrier produced in microemulsion system from aqueous extract of Aloe vera with selected antibiotics on Enterobacteriacea

**Published:** 2018-10

**Authors:** Ghasem Habibi, Mohammad Arjomandzadegan, Maryam Tayeboon, Farshideh Didgar, Hossein Sarmadian, Maryam Sadrnia, Farid Mirhosseini, Somayeh Geravand, Mahboobeh Abdoli

**Affiliations:** 1 Infectious Diseases Research Center (IDRC), Arak University of Medical Sciences, Arak, Iran; 2 Department of Biology, Payame Noor University, Tehran, Iran; 3 Department of Chemistry, Faculty of Sciences, Arak University, Arak, Iran

**Keywords:** Aloe vera, Extract, Antibacterial, Enterobacteriacea

## Abstract

**Background and Objectives:**

Antibiotics resistance has recently increased. The aim of this study was the evaluation of antibacterial efficacy of Aloe vera carrier produced in microemulsion system in comparison with ordinary antibiotics against some Enterobacteriacea.

**Materials and Methods:**

The aquatic extract of Aleo vera was produced by the Soxhlet method and a nonocarrier in the microemulsion system was prepared by two emulsifiers. The clinical isolates of *Escherichia coli, Klebsiella pneumoniae, Salmonella enterica, Shigella dysenteriae, Salmonella Typhimurium, Salmonella Paratyphi, Serratia marcescens, Proteus mirabilis, Enterobacter aerogenes, Citrobacter freundii* and *Morganella morganii* were obtained from patients and were identified by microbiological methods. Diffusion disk was used for evaluation of antibacterial properties in comparison with selected ordinary antibiotics. Minimum Inhibitory Concentration (MIC) and Minimum Bactericidal Concentration (MBC) for tested materials were determined using MTT in the Micro Broth dilution method.

**Results:**

The results proved that effect of carrier on studied isolates is dependent on concentration level. The inhibitory effect of carrier in concentration of 15 μg/ml by 18 mm zone of inhibition for *Klebsiella pneumoniae* was comparable to Ceftazidime and Cefalothin. The lowest MIC and MBC determined by the Microbroth dilution method with MTT belonged to *Klebsiella pneumoniae* as 0.1 and 3 μg/ml and higher concentrations belonged to *Enterobacter aerogenes* at 7 and 15 μg/ml. The greatest effect of carrier of Aleo vera aquatic extract was observed for *Klebsiella pneumoniae* and the lowest effect belonged to *Enterobacter aerogenes, Citrobacter freundii* and *Morganella morganii.*

**Conclusion:**

It was concluded that the carrier of Aloe vera produced in microemulsion system was most effective and had equal effects in comparison with ordinary antibiotics against Enterobacteriacea.

## INTRODUCTION

In recent years, because of the growth of resistance strains, many antibiotics have lost their efficiency. Antibiotic resistance and multi-drug resistance is a major problem in the world. As the length of patients’ hospitalization in the hospitals increases, the resistant strains are developed among populations (1–2). In addition, severe allergic reactions and immune suppression might be caused by antibiotic consumption. Therefore, it is necessary to produce natural anti-microbial drugs to cure infectious diseases. These drugs can be made of different sources, such as herbals (3).

The twenty-first century has been called the “century of using herbs to cure disease”. There are a lot of developments in research related to herbs and their curative power and every day many herbal drugs are being introduced. It has been proven that using the complete extract of herbs, instead of concentrate substance, strengthens its curative power and reduces its side-effects, because the concentration of substance is higher in the herbs. These extracts are produced in various forms, including tablet, capsule, ointment, syrup (4).

Aloe Vera has a long history, which is as old as the history of human civilization. Throughout history, it has been used as a favorite local drug. This plant is a type of Liliaceous, which has 4,000 species (5, 6). Aloe vera has two main liquid sources: yellow latex (exudates) and lucid gel (Mosilage) (4–13).

The dried latex taken from Aloe vera leaves (curacao aloe) is a combination of Aleoin, Aleo emodin, and phenol. Some phenols, including Anthraquinone and Glycoside, have been identified as active curative drugs. Over 75% of active constituent substances have been made of Aloe vera internal gel, including vitamins, enzymes, minerals, lingins, saponines, carbohydrates, sterols, amino acids, salicylic acid, etc. The effective substance of anthraquinone has been recognized as anti-bacterial, anti-viral, and anti-fungal material (14).

Enterobacter cloacae are one group of microorganisms that are involved in hospital infections. This group is one of the commonest negative Gram bacilli that have been cultured in hospitals and might cause some diseases (15). Enterobacteriaceae are a big group of Gram negative bacilli that exist naturally in the intestine of humans and animals. There are several species (*Escherichia coli, Klebsiella pneumoniae, Salmonella enterica*, *Shigella dysenteriae, Salmonella Typhimurium, Salmonella Paratyphi, Serratia marcens, Proteus mirabilis, Enterobacter aerogenes, Citrobacter freundii,* and *Morganella morganii*). They belong to the Enterobacteriaceae family. These bacteria, like *Staphylococcus* and *Streptococcus*, might cause diseases in humans (15). *Klebsiella pneumoniae* are normal flora of mouth and intestinal and can be found in the digestive system of healthy people, even in newborns (16).

In the recent years, it has been revealed that *K. pneumoniae* cause a lot infections. The importance of this group of organisms, which might cause serious infections among hospitalized patients, has been demonstrated (17–18). The capability of this organism to create diseases is very high among hospitalized patients because host defenses are decreased (as a result of complicated surgeries and the consumption of various drugs) (19–20). In a study conducted in China between 1996 and 2002, level of sensitivity to imipenem in *K. pneumoniae* was 94–100% (21).

Using nano-biotechnology to produce anti-microbial substances is a new way of research. As the concentration of the used substance is decreased, its side-effect is decreased and it becomes more economical. Microemulsion is a stable and lucid thermodynamic system that is made of water, oil, and surfactant. Because in this system the oil phase (herbal extract) is dispersed in a water enriched phase, the system is more economical and can be consumed orally (22). The aim of this study was to investigate the antibacterial characteristics of carriers of Aloe vera aquatic extract produced in microemulsion system in comparison with ordinary antibiotics.

## MATERIALS AND METHODS

### Bacterial strains.

Based on aims of the study, attempts were made to evaluate the effect of experimental materials on strains isolated from patients. Strains of *E. coli, K. pneumoniae, S. enterica, S. dysenteriae, S.* Typhimurium, *S. Paratyphi, S. marcescens, P. mirabilis, E. aerogenes, C. freundii* and *M. morganii* were obtained from patients. The clinical isolates were identified by ordinary microbiological and biochemical methods.

### Aloe vera.

Fresh Aloe vera leaves were taken from herb farm. The best Aloe vera leaves were selected and washed with sterilized distilled water and were sterilized by alcohol %70 (Ethanol). The leaves were grinded completely by a grinder.

### Extract preparation.

Extraction was done by the reflex equipment in a simple distillation method. The reflex equipment consists of 1-liter balloon and a winding 40-centimeter condenser. One hundred grams of grinded leaves and 250 ml distilled water was heated and steered for 1 hour in the equipment. Then, the temperature increased gradually until it reached to boiling point and was kept fixed.

This procedure continued for 16 hours. About 100 ml herbal extract was obtained. This extract was taken from all parts of Aloe vera. A centrifuge was used for purification of the extracts.

### Carrier production.

In this investigation and the aqueous extract solubilization studies, sample was prepared in a microemulsion system including a blend of Tween-80 (polyoxyethylene sorbitan monooleate) and Span-20 (sorbitan laurate) at a fixed weight ratio of 2:3 and was stirred to obtain a homogeneous solution.

### Diffusion disk method.

Sensitivity of isolates to carrier of Aloe vera produced in microemulsion system was determined by diffusion disk method.

Serial concentrations of extract as 40, 20, 10, 5, 2.5, 1.25, 0.62, 0.31, 0.15, 0.07, 0.035, 0.017, 0.008 mg/mL were prepared for disk diffusion method. The concentration levels of Aloe vera aquatic extract in carrier began from 2 mg/mL 8.96 μg/ml.

The strains were cultured in Mueller Hinton agar and concentration 1.5 × 10^8^ cfu/ml was obtained by OD 0.08–0.1 (in 620 nanometer wavelength). The sterilized blank disks in 5 mm in diameter were put on surface of spread plate method cultured Mueller Hinton agar. There was a 20 mm distance between the disks. 20 μl of carrier in various concentration levels were added on blank disks.

Furthermore, ordinary antibiotics were used for drug susceptibility testing as vancomycin, clindamycin, cefotaxime, ceftizoxime, ciprofloxacin, gentamicin, cefixime, tetracycline, amikacin, and co-trimoxazole were used for comparing the effect of carrier.

After 24 hours incubation at 37°C, the diameter of inhibitory zone was measured. The results of antibiotics were compared with recommendations of National Committee for Clinical Laboratory Standards (NCCLS).

### Micro broth dilution method.

The minimum inhibitory concentration (MIC) experiment was conducted on a sterilized 96-cell plate by broth micro dilution method (23–25).

In order to determine the minimum inhibitory concentration (MIC), 1.5 × 10^8^ CFU/ml of each isolate was prepared in broth Mueller Hinton from fresh colonies. 100 micro liter of the suspension was added to each well of 96-well micro plate with flat-bottom U-shaped wells. 100 micro liter of tested extract or carrier was added to the first well in each row of the first, second and up to 9^th^ column. The dilution procedure was continued to the ninth cell and 100 micro liters was taken out. It means that in 1^st^ to 9th wells, the concentrations of Aloe vera carrier were 0.5, 0.25, 0.125, 0.062, 0.031, 0.015, 0.007, 0.003, 0.001, 0.0005 etc mg/mL respectively.

In this method, the positive control method was the suspension of bacterium in broth Mueller Hinton medium without the presence of herbal extract and negative control was herbal extract and Mueller culture medium without the presence of bacterium. The 10^th^ well included 100 μl culture medium containing bacteria as positive control. The 11^th^ well included 100 μl pure carrier of Aloe vera. The 12^th^ well contained MTT without any bacteria.

For 18–20 hours, the 96-well plates were incubated in a shaker-incubator at 37°C. Turbidity of each well was determined by an ELISA reader (Stat Fax 2100 model, wavelength=550 nanometer).

Minimum inhibitory concentration was determined by a comparison between the turbidities of the wells. The lowest level of concentration of the last well which had no turbidity was considered as MIC.

### Minimum bacterial concentration (MBC).

(MBC) can be determined by micro-plate dilution method on the sterilized 96-cell plate and also through color method by MTT (26–28).

In ordinary MBC experiment, those wells which lacked turbidity were cultured separately on Mueller Hinton agar environment. After 24 hours, the level of extract concentration in which no bacteria had grown was taken as the minimum bacterial concentration.

In this study, color method by MTT was used of determination of MBC by ELISA.

The MTT assay is a colorimetric assay for assessing cell viability. NAD (P) H-dependent cellular oxidoreductase enzymes can reflect the number of viable cells present. Oxidoreductase enzyme reduces the tetrazolium dye [MTT 3-(4, 5-dimethylthi-azol-2-yl)-2,5-diphenyltetrazolium bromide] to insoluble formazan, which has a purple color.

In this method, first 20 μl MTT was added to each well and micro-plates were incubated at 37°C for 1–1.5 hours. Then 20 μl MTT color (in 5 mg/ml PBS) was added to each cell. In order to metabolize the MTT substance by living cell resulting in create Formazan crystals, the 96-cell plate was incubated at 37°C for 1 hour. When the colorful crystals were deposited, the contents of the cells was taken out and 100 μl DMSO (Di Methyl sulfoxide) was added to each cell. DMSO is solvent of formazan crystals. The addition of DMSO creates a range of colors from purple to white. The intensity of the color of these solutions is criterion for determining the number of living bacteria. The amount of cells absorption was measured by ELISA reader (wavelength=550 nm). In this method, the lack of formazan crystals in wells was due to the lack of living bacteria. So, the extract concentrations in the last well which had no formazan crystal were taken as MBC. In this method, the positive control method was the suspension of bacterium in broth Mueller Hinton without herbal extract and negative control was herbal extract and Mueller culture without bacteria.

## RESULTS

### Results of disk diffusion method.

In this study, the effect of standard disks of vancomycin, clindamycin, cefotaxime, ceftizoxime, ciprofloxacin, gentamicin, cefixime, tetracycline, amikacin, and co-trimoxazole on clinical strains of *E. coli, K. pneumoniae, S. enterica, S. dysenteriae, S.* Typhimurium, *S. Paratyphi, S. marcescens, P. mirabilis, E. aerogenes, C. freundii* and *M. morganii* were determined (Table 1).

Results of inhibition zones in this method were compared with results of carrier of Aloe Vera (Table 2).

**Table 1.** The results of drug susceptibility testing of studied strains

**Antibiotics**	**Tetracycline TE 30 μg**	**Ceftazidime CAZ 30 μg**	**Ciprofloxacin CIP 5 μg**	**Streptomycin Ampicillin STR 10 μg**	**Gentamicin AM 10 μg**	**Cefalothin GEN 10 μg**	**CEF 30 μg**

**strains**							
*Escherichia coli*	S	R	S	R	-	S	I
	22	15	32	15			15

*klebsiella pneumoniae*	S	R	S	R	-	S	I
	22	15	33	11			15

*Salmonella enterica*	-	-	S	-	I	R	R
			30		17	18	9

*Shigella dysenteriae*	R	-	S	-	R	-	R
			22				10

*Salmonella Typhimurium*	-	R	S	-	-	-	S
		15	33				16

*Salmonella Paratyphi*	I	R	S	-	-	S	I
	15	15	40				17

*Proteus mirabilis*	R	R	S	13	R	I	S
		15	35		13	13	22

*Enterobacter aerogenes*	S	R	I	R	-	-	R
	17		18				

*Citrobacter freundii*	-	S	R	R	R	-	I

*Morganella morganii*	S	R	I	R		-	R

**Table 2.** The results of carrier effects on studied strains in diffusion disk method (diameter of zone of inhibition, mm)

**Extract dilution**	**1**	**1/2**	**1/4**	**1/8**	**1/16**	**1/32**	**1/64**	**1/128**	**1/256**
Extract (mg/ml)	0.5	0.25	0.125	0.062	0.031	0.015	0.007	0.003	0.001
Strains									
*Escherichia coli*	30	28	25	20	18	12	10	-	-
*klebsiella pneumoniae*	40	30	28	25	20	18	12	8	-
*Serratia marcescens*	35	32	30	28	25	18	12	8	-
*Salmonella enterica*	22	18	17	15	12	10	8	-	-
*Shigella dysenteriae*	22	20	15	10	-	-	-	-	-
*Salmonella Typhimurium*	30	28	25	18	12	10		-	-
*Salmonella Paratyphi*	32	24	23	22	17	13	-	-	-
*Proteus mirabilis*	30	28	28	25	24	17	-	-	-
*Enterobacter aerogenes*	18	10	-	-	-	-	-	-	-
*Citrobacter freundii*	25	20	10	-	-	-	-	-	-
*Morganella morganii*	30	25	22	15	10	-		-	-

Results of anti-microbial effects of carrier on clinical strains of *E. coli, K. pneumoniae, S. enterica, S. dysentery, S.* Typhimurium, *S. Paratyphi, S.marcens, P. mirabilis, E. aerogenes, C. freundii* and *M. morganii* has been presented in Table 2. Concentrations of carrier in Table 2 were calculated as pure content of dry weight of extract in carrier.

As shown in Table 1, *K. pneumoniae* has a 15 mm zone of inhibition for ceftazidime (30 μg) that was marked as resistant. This bacterium was resistant to streptomycin (10 μg) by 11 mm but showed a 20 mm zone in 31 μg and 8 mm in 3 μg of carrier (Table 2). This situation was the same for other antibiotics for this bacterium such as ciprofloxacin, cefalothin, gentamicin etc. That means carrier could inhibit *K. pneumoniae* better than ordinary antibiotics.

Antibiotics disks used against *E. coli* have various concentrations. For example 5 μg for ciprofloxacin to 30 μg for tetracycline, ceftazidime and cefalothin with zones of inhibition as follows; 32 mm, 22 mm, 15 mm and 15 mm, respectively. Those would be compared by good reactions of carrier with zones of inhibition as 10 mm to 20 mm for 7 μg to 62 μg, respectively.

**Fig. 1.**
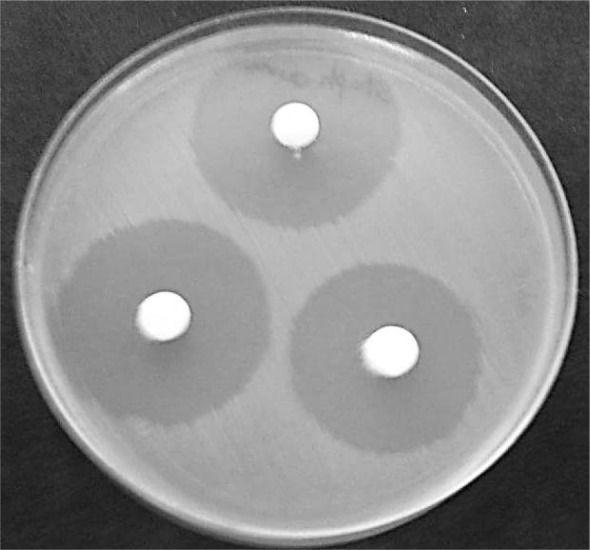
Kirby-Bauer’s results for concentrations of nanocarrier of Aloe vera extract for *Klebsiella pneumoniae*. A: 7 μg/ml, B:15 μg/ml, C: 31 μg/ml

**Fig. 2.**
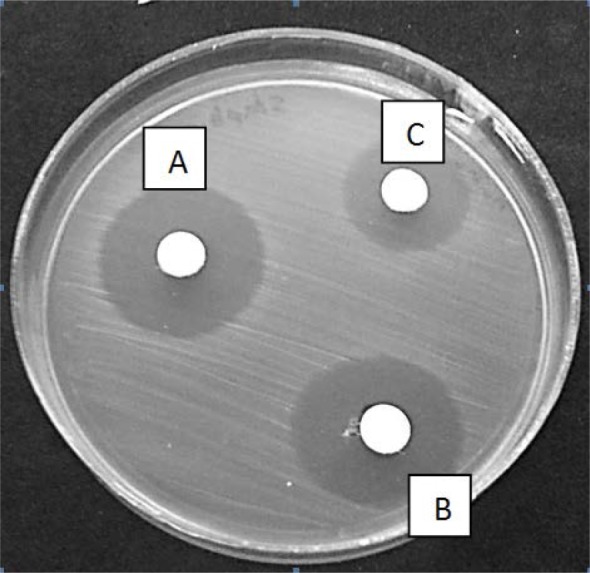
Kirby-Bauer’s results for concentrations of carrier of Aloe vera extract in *Salmonella Typhimurium.* A: 62 μg/ml, B:31 μg/ml, C: 15 μg/ml

As shown in Table 2, the worst susceptibility to carrier of Aleo vera extract belonged to *E. aerogenes* and *C. freundii*. The carrier had the maximum effect on the clinical strain of *K. pneumoniae* (Figs 1 and 2).

The results of measuring turbidity by Micro-Broth dilution method showed that they were almost consistent with the results of diffusion disk method given in Tables 2 and 3.

**Table 3.** The results of diffusion disk method of the studied straions

**Clinical Isolates**	**MBC μg/ml**	**MIC μg/ml**
*Klebsiella pneumoniae*	3	0.1
*Serratia marcescens*	3	0.1
*Escherichia coli*	7	0.2
*Salmonella enterica*	7	0.2
*Shigella dysenteriae*	7	3
*Salmonella* Typhimurium	3	0.5
*Salmonella* Paratyphi	3	0.5
*Proteus mirabilis*	3	0.5
*Enterobacter aerogenes*	15	7
*Citrobacter freundii*	15	7
*Morganella morganii*	15	1

MIC was 0.1 μg/ml for both *K. pneumoniae*, *S. marcens* and was 0.2 μg/ml for both *S. enterica* and *E. coli*. MBC for these strains was 3 and 7 μg/ml, respectively.

*S. dysenteriae* was inhibited in higher concentrations of extraction in 3 μg/ml but *S. Typhimurium* in 0.5 μg/ml with a MBC equal to 7 μg/ml and 3 μg/ml, respectively.

*E. aerogenes, C. freundii* and *M. morganii* have the largest MIC and MBC for carrier and showed a higher resistance that was equal to reaction of these strains to carrier in Disk Difusion Method (Tables 2 and 3).

## DISCUSSION

Because various bacteria are resistant to a wide range of antibiotics, a lot of attempts have made to cure human disease by herbs. In Iran, a lot of studies have been conducted on the characteristics of Aloe Vera, but the studies on anti-microbial characteristics of Aloe vera and its effect on clinical bacteria have been limited. In 2012, Fani studied the inhibitive effect of Aloe vera on cancer and also the damaging effects of several bacteria on the gum (29). In this study, the anti-bacterial characteristics of Litoralis Aloe vera extract was demonstrated.

Agarry (2005) conducted a comparative study of the effect of fiber and gel of Aleo vera on bacteria such as *S. aureus, P. aeruginosa, T. mentagraphytes, T. schoeleinii, M. canis* and *C. albicans* (30). Based on the results of this study, the use of both fibers and leaves was recommended. In this study, it was demonstrated that the extract taken from complete leaves had a major anti-microbial effect on clinical strains. So, the complete extract of Aloe vera was used to produce carrier. George (2008) studied the effect of Aloe vera tooth paste on *C. albicans, S. mutans, L. acidophilus, E. faecalis, P. intermedia* and *P. anaerobius* and showed the positive anti-microbial characteristics of tooth paste (31). In this study, the maximum zone of inhibition was 40 mm for carrier of Aloe vera extract in its pure form and a concentration of 8.96 μg/ml for *Klebsiella*. The minimum, in the same concentration, belonged to *Enterococcus* at 18 mm.

Compared to other bacteria, *Enterococcus* is more resistant to carrier. In the 4.48 μg/ml concentration, the last zone of inhibition growth in diameter was 8±2. The three repetition mean of the zone of inhibitory growth in diameter has been presented in Table 2. A comparison between disk diffusion method and chromatography showed that the sensitivity of micro-plate method for determining the minimum inhibitory concentration is higher. The carrier of Aloe vera extract, which has an effect on bacteria with a very lower concentration, is suggested for curative proposes.

In conclusion, The carrier of Aloe vera had an effect on the bacteria with very low concentration and it is suggested for curative purposes.
